# Trends and age-period-cohort analysis of bacteriologically confirmed pulmonary tuberculosis: a population-based study in Hunan Province, China, 2009–2023

**DOI:** 10.3389/fpubh.2026.1755269

**Published:** 2026-03-31

**Authors:** Guojun Huang, Qiqi Wang, Wenjing Zheng, Jianjun Liu, Liqiong Bai, Jun Liang, Zuhui Xu, Shicheng Yu, Yanping Wan, Xie Liu, Hongyan Yao, Hui Ming

**Affiliations:** 1 Chinese Center for Disease Control and Prevention (Chinese Academy of Preventive Medicine), Beijing, China; 2 Hunan Institute for Tuberculosis Control (Hunan Chest Hospital), Changsha, Hunan, China

**Keywords:** age-period-cohort model, China, influencing factors, joinpoint regression, pulmonary tuberculosis, seculartrends

## Abstract

**Background:**

Trends in the incidence of bacteriologically confirmed pulmonary tuberculosis (PTB), a primary source of transmission, are important for targeted prevention and control. Hunan Province is a high-PTB-burden region in China. However, conventional trend analyses cannot separate the independent effects of age, period, and birth cohort, and this question has not been specifically examined in Hunan Province.

**Methods:**

Using PTB surveillance data from Hunan Province (2009–2023), this study combined joinpoint regression and an age-period-cohort (APC) model to assess long-term trends in the reported incidence of bacteriologically confirmed PTB and quantify the independent effects of age, period, and birth cohort.

**Results:**

Over the study period, the age-standardized reported incidence of bacteriologically confirmed PTB declined (average annual percent change [AAPC], −2.20%). This decrease was more marked among males (AAPC, −2.54%), whereas the decline among females was not statistically significant. The decline plateaued after 2017, with an inflection point in 2020. APC analysis indicated a bimodal age pattern, with peaks in the 20–24 and 80–84-year age groups. The increase in incidence with age was greatest in the oldest age group. The period effects were statistically significant. The post-2017 plateau may be associated with changes in diagnostic practices and/or reporting, and the 2020 inflection point coincided with the COVID-19 pandemic. Cohort effects showed that risk peaked in the 1949–1953 birth cohort and then declined steadily; however, among females, cohorts born after 1994 showed early indications of a possible increasing risk.

**Conclusion:**

APC analysis of bacteriologically confirmed PTB in Hunan Province showed a shifting epidemiology. These findings suggest that tuberculosis control efforts should prioritize the increasing burden in older adults and closely monitor younger female cohorts for possible increases in risk. These results may help refine interventions for high-risk groups and optimize surveillance strategies.

## Introduction

Pulmonary tuberculosis (PTB) remains a major public health problem. According to the World Health Organization, in 2023, 6.9 million people were reported to have been diagnosed with PTB worldwide, of whom 62% were bacteriologically confirmed (1). Bacteriologically confirmed PTB poses a substantial public health risk because of its transmissibility; one individual with smear-positive PTB is estimated to infect 8–10 other individuals annually (2). Inadequate or incomplete treatment of smear-positive PTB can contribute to the development of drug-resistant tuberculosis (TB), thereby increasing the risk of clustered outbreaks. From a public health perspective, bacteriologically confirmed PTB plays a crucial role in transmission (3, 4). Accordingly, the *2021 Technical Guidelines for Tuberculosis Prevention and Control in China* (5) identify bacteriologically confirmed cases as key targets for management and recommend strengthened control measures, including case isolation and active screening of close contacts. Therefore, detailed analyses of trends in bacteriologically confirmed PTB can provide deeper insights into its epidemiologic patterns and inform evidence-based prevention and control measures.

Surveillance data indicate that the reported incidence of PTB has been continuously decreasing in several provinces of China. However, the incidence of bacteriologically confirmed PTB has not shown a concomitant decrease and has gradually increased in some regions (6–8). This divergence suggests that bacteriologically confirmed PTB may have distinct epidemiologic drivers. Conventional trend analyses, such as joinpoint regression, can describe temporal patterns but are limited in identifying underlying mechanisms, specifically, whether observed changes reflect population aging (age effect), public health interventions and contextual changes (period effect), or generational differences (cohort effect).

The age-period-cohort (APC) model provides an analytical framework to address this limitation. By disentangling these three effects, the APC model has been widely applied in studies on cancer (9, 10) and infectious diseases (11, 12). In China, research has demonstrated independent associations of age, period, and birth cohort with PTB incidence (13). However, APC studies have not specifically focused on bacteriologically confirmed PTB as an important subgroup. This is a meaningful knowledge gap, because the epidemiology of bacteriologically confirmed PTB may differ from that of overall PTB. First, its trends may reflect the transmission potential more directly. Second, the proportion of cases that are bacteriologically confirmed is a core indicator of program performance and may be influenced by surveillance bias introduced by advances in diagnostic technologies (e.g., rollout of molecular testing). The APC model is particularly suited for quantifying period-related changes that may reflect such diagnostic and surveillance shifts, separate from the underlying epidemiologic changes.

In China, PTB incidence varies by province (14). Hunan Province is a high-TB-burden region (15); however, trends in bacteriologically confirmed PTB have not been systematically examined across age, period, and cohort dimensions. Therefore, using TB surveillance data in Hunan Province from 2009 to 2023, this study aimed to: (1) describe long-term trends in the reported incidence of bacteriologically confirmed PTB using joinpoint regression; (2) quantify the independent effects of age, period, and birth cohort on bacteriologically confirmed PTB incidence using an APC model; (3) identify high-risk populations and key periods, including sex-specific differences; and (4) examine the potential contribution of improved diagnostic capacity to observed surveillance trends by integrating joinpoint inflection points with estimated period effects, thereby providing an evidence base for refining and optimizing TB prevention and control strategies.

## Methods

### Data sources

Information on reported PTB cases and reported incidence in Hunan Province, stratified by period, sex, and age group, was extracted from the China Disease Prevention and Control Information System. The population denominators were obtained from the same surveillance system for each year of the study period. The annual population is categorized by 5-year age groups and sex. The year-end resident population estimates for the specific year were used as the population count. PTB diagnoses were defined according to the National Health Department standards *Diagnosis of Pulmonary Tuberculosis (WS 288-2008)* before May 1, 2018, and according to *Diagnosis of Pulmonary Tuberculosis (WS 288-2017)* thereafter. Case classification followed the *Tuberculosis Classification (WS 196-2017)*. According to the notification on adjustments to PTB reporting categories (16), cases of bacteriologically confirmed PTB, including cases of rifampicin-resistant PTB, were defined as cases of PTB with smear-positive, culture-positive, or positive molecular test results. Cases of laboratory-negative PTB and cases with no TB laboratory results available were excluded from the analysis. TB prevention and control agencies at all administrative levels routinely conduct checks for under-reporting and missed registrations to improve the completeness and accuracy of case reporting and registration.

### Statistical analysis

#### Age-standardized reported incidence of bacteriologically confirmed PTB

To control for differences in population age structure across years, the age distribution from the Sixth National Population Census of China (2010) was used as the standard population. The age-standardized reported incidence of bacteriologically confirmed PTB was calculated using the direct standardization method as follows:


$$\begin{array}{r} Age-standardised\;reported\;incidence\;of\;bacteriologically\;\\ confirmed\;PTB=\frac{\text{Σ}N_{i}P_{i}}{N} \end{array}$$

where *N*_*i*_ represents the i^th^ age category in the reference population, *P*_*i*_ represents the actual reported incidence in the i^th^ age category, and *N* represents the total reference population.

#### Joinpoint regression model

Trends in the reported incidence of bacteriologically confirmed PTB in Hunan Province from 2009 to 2023 were evaluated using joinpoint regression (17, 18). The annual percent change and average annual percent change (AAPC) were estimated to assess whether the trends within segments were statistically significant. If the confidence interval for the annual percent change or AAPC did not include 0, it denoted a statistically significant change in that segment; otherwise, it suggested a lack of statistically significant change. The natural logarithm of the reported incidence of bacteriologically confirmed PTB was modeled as the dependent variable, with the calendar year as the independent variable. The maximum number of joinpoints was set to 4; the inflection points were identified using the grid-search method; and the optimal model was selected using the weighted Bayesian information criterion. Analyses were conducted using the Joinpoint Regression Program (version 5.4.0.0; National Cancer Institute, Bethesda, MD, USA; released in April 2025).

#### APC model

An APC model was used to estimate the independent effects of age, period, and birth cohort on trends in the reported incidence of bacteriologically confirmed PTB. In this framework, the age effect reflects variation in incidence by age group; the period effect reflects changes over calendar time that affect all age groups (e.g., screening practices, diagnostic technology, and reporting/classification criteria); and the cohort effect reflects differences across birth cohorts potentially related to long-term changes in exposures and risk factors (19). As the available population data were structured in 5-year age groups, we adopted corresponding 5-year periods to maintain internal validity.

Bacteriologically confirmed PTB cases were grouped into 17 5-year age groups (0–4, 5–9, …, 75–79, and 80–84 years). Those aged ≥85 years were excluded because 5-year age group data were not available. The study period (2009–2023) was grouped into three 5-year calendar periods (2009–2013, 2014–2018, and 2019–2023). Birth cohorts were grouped into 19 5-year cohorts (1929–1933, 1934–1938, 1939–1943, …, and 2019–2023). The linear dependency among age, period, and cohort is expressed as: cohort = period – age. For example, patients aged 10–14 and 15–19 years diagnosed during the period 2009–2013 correspond to the birth cohorts of 1995–2003 and 1990–1998, respectively, and in this analysis were included in the 1999–2003 and 1994–1998 birth cohorts, respectively.

Because APC models have an identifiability problem (i.e., parameters do not have a unique solution), analyses were conducted using the APC analysis tool provided by the US National Cancer Institute (https://dceg.cancer.gov/tools/analysis/apc), which has been used in previous studies (20). In this study, the middle category was used as the reference group for the period (2014–2018) and the birth cohort (1974–1978), according to the default settings of the tool. We used the longitudinal reported age-specific PTB incidence to assess the age effect, with no reference group. The statistical significance of the parameters and functions was evaluated using the Wald χ^2^ test. All tests were two-tailed, and *P-*values < 0.05 were considered statistically significant. Figures were generated using R version 4.3.1 (R Foundation for Statistical Computing, Vienna, Austria).

## Results

### Reported incidence of bacteriologically confirmed PTB in Hunan Province

**Figure 1:**
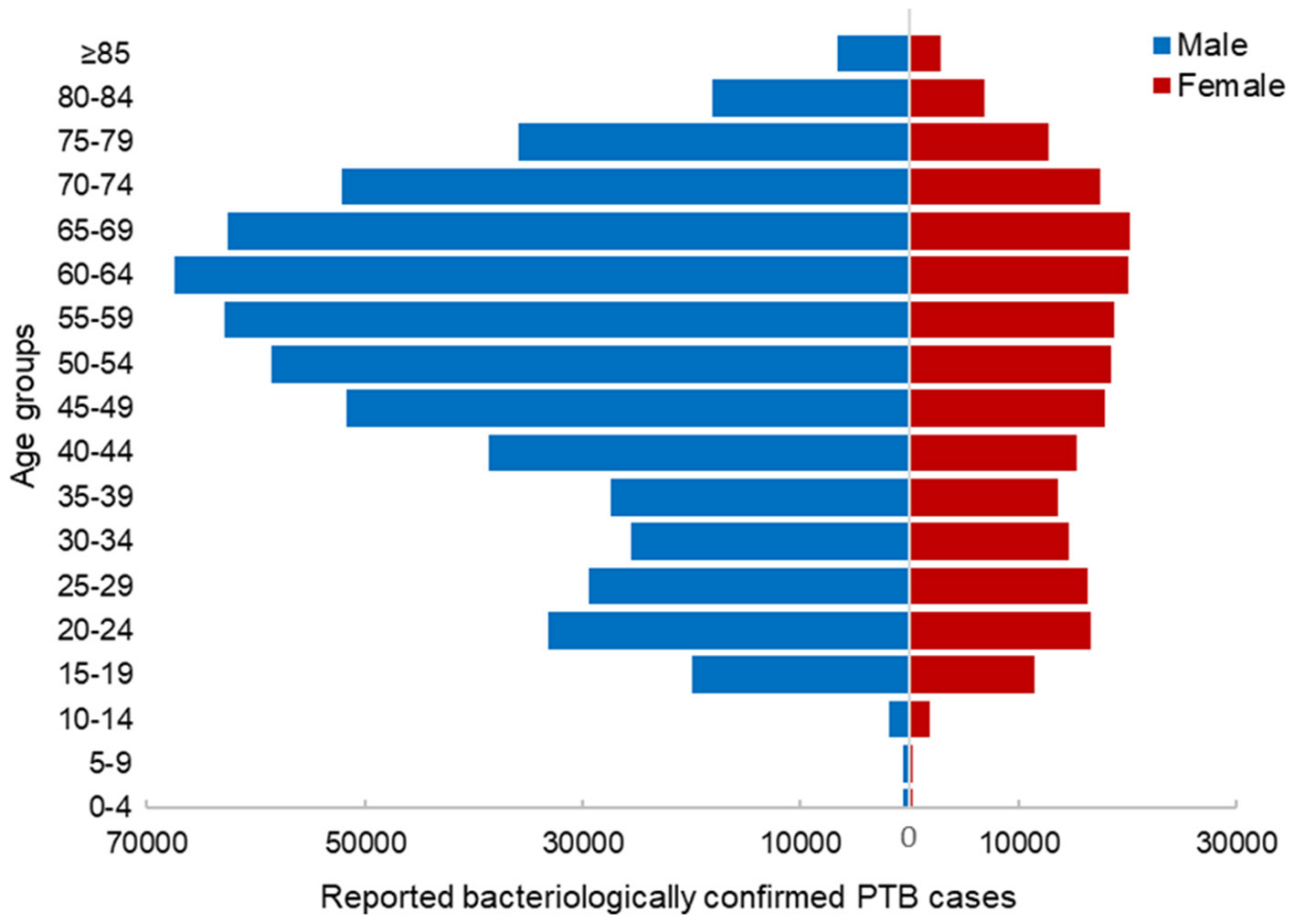
Number of reported cases of bacteriologically confirmed pulmonary tuberculosis in Hunan Province, China, 2009–2023 according to age group and sex.

From 2009 to 2023, a total of 361,792 cases of bacteriologically confirmed PTB were reported in Hunan Province, with an average annual reported incidence of 36.12 per 100,000 population (range, 26.51–42.54 per 100,000 population). Among these patients, 268,150 (74.12%) were male, and 93,642 (25.88%) were female. The highest number of cases (39,730) occurred in the 60–64-year age group, accounting for 10.98% of the total number of cases reported. The number of reported cases was higher in males than in females in all age groups, except the 10–14-year age group (Figure 1).

### Joinpoint regression results

Between 2009 and 2023, the age-standardized reported incidence of bacteriologically confirmed PTB in Hunan Province showed an overall declining trend; however, this decline has slowed since 2017. Moreover, this decrease was primarily driven by males, whereas the reported incidence among females did not show a significant reduction. Joinpoint regression analysis showed that the age-standardized reported incidence of bacteriologically confirmed PTB in Hunan Province declined from 2009 to 2023 (AAPC: −2.20%; 95% CI: −3.22% to −1.39%). Inflection points were identified in 2017 and 2020. The average annual decline from 2009 to 2017 was −6.83% (95% CI: −9.20% to −5.20%). Fluctuations were observed from 2017 to 2020 and from 2020 to 2023; however, these fluctuations were not statistically significant. The age-standardized reported incidence of bacteriologically confirmed PTB stratified by sex remained consistently higher in males than in females. The decline in age-standardized reported incidence was more rapid in males (AAPC: −2.54%; 95% CI: −3.86% to −1.45%) than in females (AAPC: −0.99%; 95% CI: −2.33% to 0.04%), and the change in incidence in females was not statistically significant (Table 1).

**Table 1:** Joinpoint analysis results of the age-standardized reported incidence of pulmonary tuberculosis in Hunan Province (2009–2023) by sex.

Category	No. of inflection points	Period	Annual percent change (95% CI)	AAPC (2009–2023) (95%CI)
All	2	2009–2017	−6.83^*^ (−9.20, −5.20)	−2.20^*^ (−3.22, −1.39)
		2017–2020	12.30 (−3.07, 16.42)	–
		2020–2023	−3.07 (−12.35, 3.38)	–
Male	2	2009–2017	−7.20^*^ (−10.96, −4.15)	−2.54^*^ (−3.86, −1.45)
		2017–2020	11.77 (−9.30, 16.88)	–
		2020–2023	−3.15 (−14.78, 4.32)	–
Female	2	2009–2017	−5.73^*^ (−9.08, −3.43)	−0.99 (−2.33, 0.04)
		2017–2020	14.14 (−6.79, 19.19)	–
		2020–2023	−2.16 (−13.72, 5.73)	–

AAPC, average annual percent change.

^*^Indicates P < 0.05.

### APC analysis of the reported incidence of bacteriologically confirmed PTB

Net drift analysis showed that between 2009 and 2023, the reported incidence of bacteriologically confirmed PTB in Hunan Province decreased at an average annual rate of −1.73% (95% CI: −2.82% to −0.63%). The net drift in males was −2.19% (95% CI: −3.41% to −0.95%) and that in females was −0.45% (95% CI: −1.42% to 0.53%), indicating that the annual decline in incidence was more rapid in males than in females.

**Figure 2:**
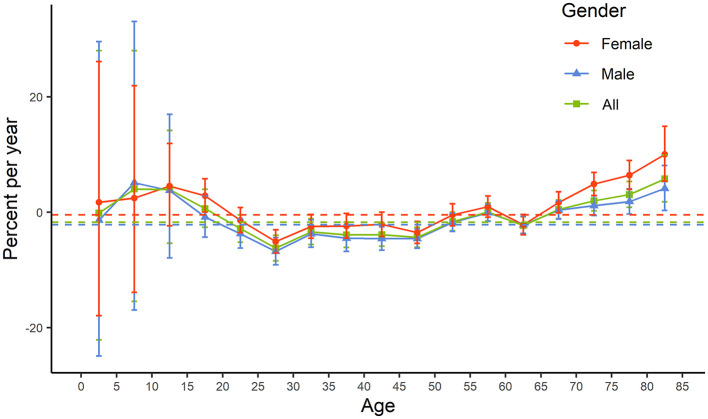
Net drift and local drift diagram. The vertical lines represent the 95% confidence intervals for local drift, whereas the horizontal dashed line indicates the net drift.

Local drift analysis revealed that the reported incidence of bacteriologically confirmed PTB in both males and females showed the most rapid average annual decrease in the 25–29-year age group, with the incidence decreasing at an average annual rate of −6.79% (95% CI: −9.11% to −4.41%) in males and −5.09% (95% CI: −7.09% to −3.05%) in females. In contrast, the most rapid average annual increase in reported incidence was observed in the 80–84-year age group in both females (10.03%; 95% CI: 5.37% to 14.88%) and males (4.11%; 95% CI: 0.29% to 8.08%) (Figure 2; Supplementary Tables S1 and S2). This indicates a concerning trend of the burden of PTB shifting toward the older adults.

**Figure 3:**
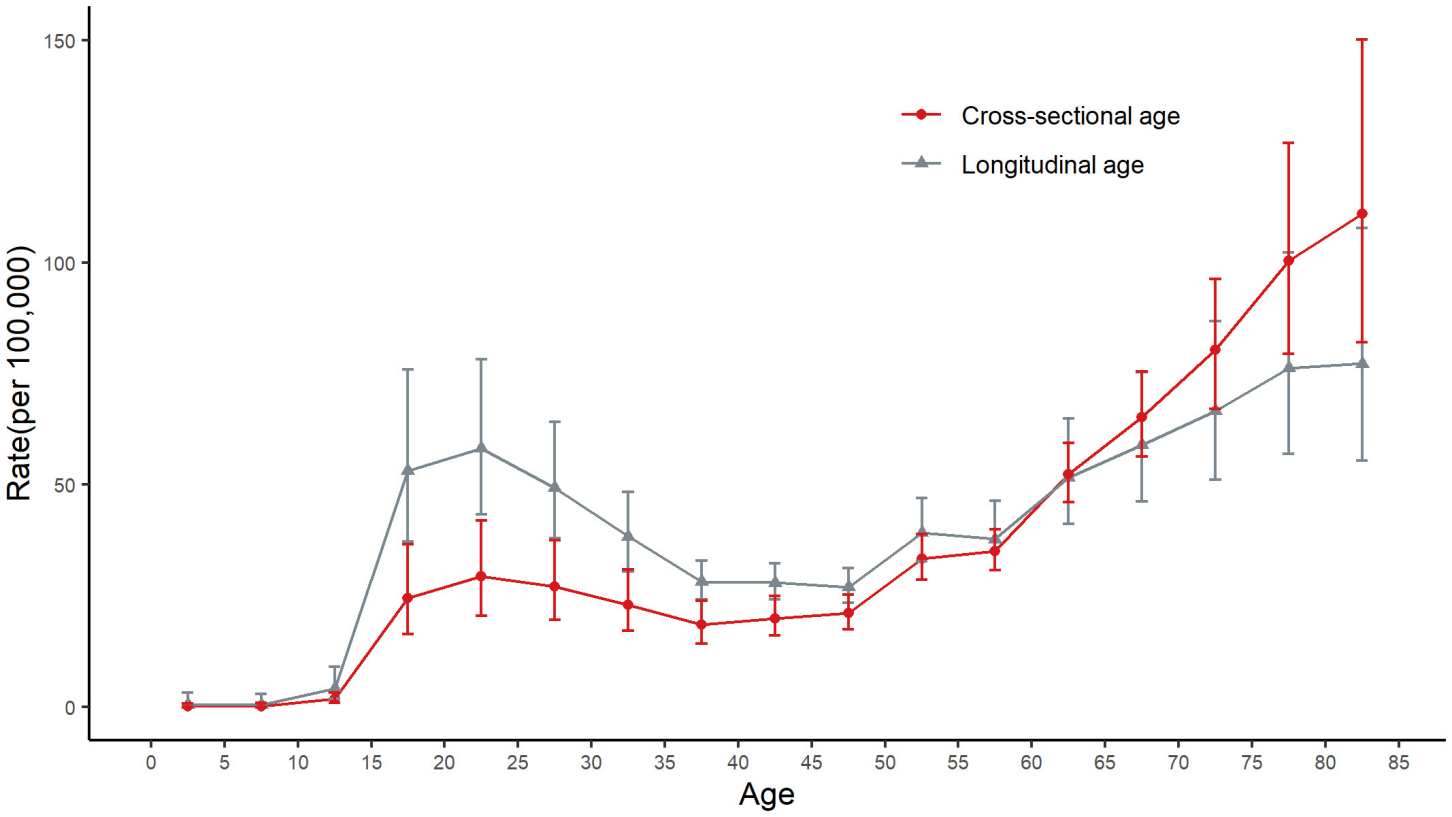
The incidence of bacteriologically confirmed PTB in Hunan Province by cross-sectional and longitudinal age groups. The vertical lines represent the 95% confidence intervals.

The separation of cross-sectional and longitudinal cohort age groups indicated that there was a cohort effect on the reported incidence of bacteriologically confirmed PTB in Hunan Province, suggesting that historical exposure or protective factors influenced PTB incidence risk across birth cohorts. In the 0–14-year age group, the incidence from both cross-sectional and longitudinal data was similar. However, in the 15–59-year age group, the cross-sectional incidence was lower than the longitudinal incidence. In contrast, in the ≥60–64-year age group, the cross-sectional incidence was higher than the longitudinal incidence (Figure 3).

#### Age effect

**Figure 4:**
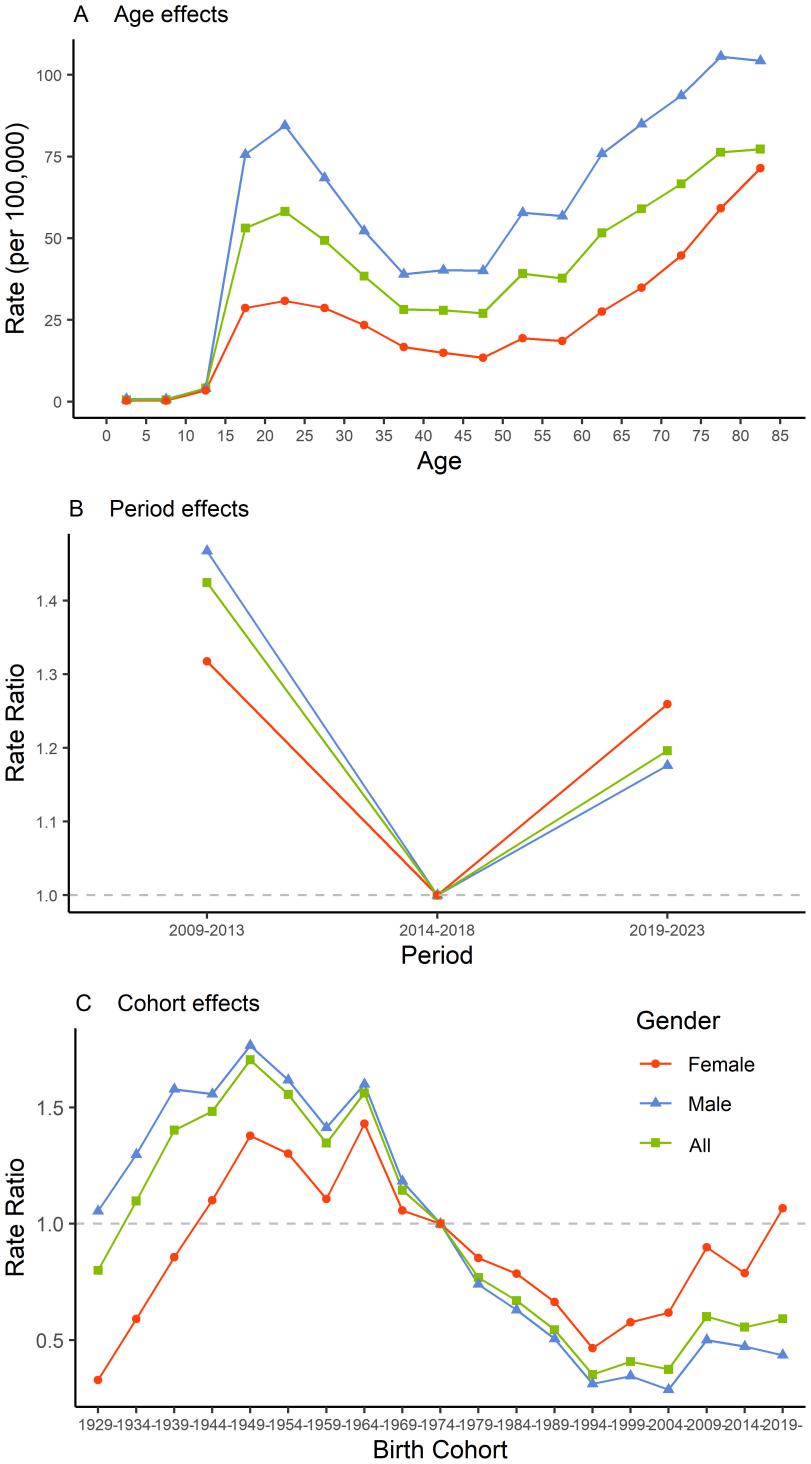
Results of the age-period-cohort analysis of the reported incidence of bacteriologically confirmed pulmonary tuberculosis in Hunan Province from 2009 to 2023. **(A)** Age effect; **(B)** Period effect; and **(C)** Cohort effect.

The reported incidence of bacteriologically confirmed PTB exhibits a distinct bimodal age distribution. The reported incidence of bacteriologically confirmed PTB was stable and low in the 0–14-year age group, increased sharply in the 15–19-year age group, reached a first peak in the 20–24-year age group, gradually decreased, reached a trough in the 45–49-year age group, and then increased again, peaking in the 80–84-year age group. Trends were similar in males and females, but the incidence was higher in males (Figure 4A).

#### Period effect

Using 2014–2018 as the reference period, the rate ratio (RR) of reported incidence of bacteriologically confirmed PTB was 1.42 (95% CI: 1.31–1.55) for 2009–2013 and 1.20 (95% CI: 1.10–1.30) for 2019–2023. In 2009–2013, the RR was higher for males (1.47; 95% CI: 1.34–1.60) than for females (1.32; 95% CI: 1.21–1.43). However, in 2019–2023, the RR was higher for females (1.26; 95% CI: 1.16–1.36) than for males (1.18; 95% CI: 1.08–1.28) (Figure 4B).

#### Cohort effect

Using the 1974–1978 birth cohort as the reference cohort, cohort analysis revealed that the RR initially increased and then decreased, peaking in the 1949–1953 birth cohort (1.70; 95% CI: 1.33–2.18). After reaching this peak, the RR fluctuated downward, with cohorts born after 1974–1978 having RRs < 1.0. However, the cohort effect for females born between 1994 and 2023 displayed a fluctuating upward trend, with an RR of 1.07 (95% CI: 0.11–10.12) in the 2019–2023 birth cohort. For cohorts born before 1974–1978, the RR was greater for males than for females; however, after 1978, the RR for females exceeded that for males (Figure 4C; Supplementary Table S3). However, these estimates should be interpreted with caution owing to the wide width of some confidence intervals.

## Discussion

This study is the first to comprehensively apply the APC model to analyze the epidemiology of bacteriologically confirmed PTB in Hunan Province. We combined joinpoint regression with APC analysis to clarify long-term trends, revealing three core findings: (1) the reported incidence of bacteriologically confirmed PTB in Hunan Province showed a declining trend, primarily driven by a large overall decrease in males, whereas the decline in females was not significant; (2) the reported incidence among the older and young populations exhibited a bimodal distribution; and (3) the RR in recent birth cohorts among females showed a fluctuating upward trend. These findings indicate that the TB epidemic is changing, necessitating adjustments in prevention and control strategies.

The reported incidence of bacteriologically confirmed PTB in Hunan Province showed a declining trend from 2009 to 2017, consistent with observations from other provinces during the same period, such as Hainan Province (21), Shaanxi Province (22), and Jiangsu Province (8). This improvement is primarily attributable to national TB prevention and control planning objectives, increased financial investment, and the implementation of prevention and control measures by health authorities at various levels, resulting in more effective control. However, after 2017, the trend shifted, with an increase in incidence that continued until 2020, which aligns with the reports from Jiangsu (8) and Liaoning (23). This rise during this phase does not necessarily imply epidemiologic deterioration; rather, it likely reflects enhanced diagnostic capabilities during the same period. Since 2017, Hunan Province has continuously promoted the construction of a TB laboratory network and has widely introduced rapid molecular diagnostic techniques (e.g., GeneXpert), improving the detection capacity for bacteriologically confirmed cases. Related studies have also confirmed that the proportion of bacteriologically confirmed PTB cases in the province rose sharply from 36.32% in 2017 to 57.74% in 2022 (7). The second turning point observed in 2020 may be related to the impact of COVID-19 prevention and control measures on the TB service system, possibly leading to delays in case detection and treatment (24, 25). In addition, these measures may have reduced the number of patients seeking medical care and receiving nursing services (26), resulting in a relatively stable plateau in the reported incidence. From a contextual perspective, these trend changes identified by joinpoint regression align with fluctuations in period effects within the APC model, suggesting that recent changes in incidence rates may be influenced not only by underlying epidemiologic changes but also by temporal factors, such as the widespread adoption of diagnostic technologies and major public health events. Specifically, this period effect may reflect the impact of socioeconomic development, updates in diagnostic standards, and the introduction of new technologies on disease surveillance and reporting outcomes. The implementation of new national standards for TB diagnosis in 2018 and the promotion of molecular testing technology likely further facilitated the identification of bacteriologically confirmed cases, highlighting the role of diagnostic advancements in shaping reporting trends.

A net drift value of −1.73% indicates an overall annual decline, and the separation of cross-sectional and longitudinal age curves suggests a birth cohort effect (20). The longitudinal age curve revealed a bimodal distribution in the incidence of bacteriologically confirmed PTB, with peaks occurring in the youth group aged 15–29 years and the older adults aged 60–84 years, while incidence in the 0–14-year age group was substantially lower. Another study conducted in China reported similar results (13). This age pattern may be related to differences in infection risk (27, 28), immune status (29, 30) (e.g., the effectiveness of the BCG vaccine waning with age), and social behavior patterns (31) at different life stages. We propose prioritizing resources for older adults (60–84 years) and recommend piloting the integration of active TB screening into community-based older adults care facilities and chronic disease management programs.

The cohort effect further illustrates intergenerational shifts in risk of acquiring TB. Overall, the RR for birth cohorts born after 1949 shows a “first increase then decrease” pattern, peaking in the 1949–1953 cohort before continuously declining, which aligns with macro-level advances such as socioeconomic development, the establishment of healthcare systems, and the implementation of the DOTS strategy (32). Notably, the increased risk in the cohort born between 1964 and 1968 may be related to historical factors, such as nutritional deficiencies experienced during childhood (33). In contrast, more recent cohorts (2014–2023), particularly female cohorts, show an upward trend in risk. Another study (34) conducted in Hunan Province during the same period (2014–2023) found an increasing TB incidence among children aged 0–14 years, with girls showing a higher growth rate than boys, which is consistent with our results. This suggests a need to monitor the potential emerging risks faced by younger cohorts.

Our study found significant heterogeneity in the reported incidence and temporal changes in bacteriologically confirmed TB by sex. In addition, the age-specific reported incidence was higher in males than in females. This could be associated with interactions between biological factors, social roles, and behaviors (35). First, males tend to engage in more outdoor activities, have broader social networks, may have greater exposure to sources of infection, and are more likely to engage in heavy physical labor with dust exposure (e.g., mining) and harmful behaviors such as smoking and high alcohol intake (35). Second, animal studies have shown that higher estrogen levels can reduce susceptibility to TB (36). Third, among individuals with latent *Mycobacterium tuberculosis* infection, the risk of progression to active TB is higher in males than in females (37). The decline in the reported incidence of bacteriologically confirmed TB was more rapid in males than in females, which may be related to the higher incidence among males, greater potential for decline, and improvements in male behavioral risk factors, including reduced smoking, alcohol consumption, and occupational exposure.

The above explanations are based on ecological data, are hypothesis-generating, and aim to provide a macro-level framework for interpreting observed APC patterns, rather than serving as causal evidence at the individual level. As a high-burden province, Hunan's epidemiologic patterns may not be directly applicable to regions with lower TB incidence or different demographic and health system profiles.

To our knowledge, this study is the first to apply the APC model to systematically analyze the epidemiologic characteristics of bacteriologically confirmed PTB. This provides insights into how age, period, and birth cohort influence the reported incidence. These findings suggest that recent advances and expanded implementation of diagnostic technologies for TB may have contributed to the increased reporting of bacteriologically confirmed PTB cases. These findings provide evidence for optimizing TB prevention and control strategies and suggest that continued diagnostic innovation and improvements in the healthcare system may help address current prevention and control challenges and enhance the effectiveness of TB control.

However, this study has some limitations. First, this is an ecological analysis; therefore, the potential for the ecological fallacy should be considered. Second, the data source was case notification data, which may have underestimated the true incidence of bacteriologically confirmed PTB. Third, this study was conducted at the population level and did not account for individual health conditions, ethnicity, or socioeconomic and environmental factors. Future studies should consider including additional potential influencing factors. Finally, excluding individuals aged ≥85 years may lead to an underestimation of PTB burden and limit inferences regarding cohort-specific risks within this high-risk demographic.

APC analysis of bacteriologically confirmed PTB in Hunan Province indicated a shifting epidemiology. Although the overall reported incidence declined (primarily among males), progress plateaued after 2017. The disease shows a persistent bimodal age distribution, with the steepest increase in older age groups and an increasing RR in more recent female birth cohorts. These findings indicate that current control efforts should prioritize the growing burden among older adults and closely monitor potential risk changes in younger female cohorts. The results provide evidence to refine interventions for high-risk groups and optimize surveillance systems.

## Data Availability

The original contributions presented in the study are included in the article/Supplementary material, further inquiries can be directed to the corresponding authors.
