# Supplementary material for: Trends and age-period-cohort analysis of bacteriologically confirmed pulmonary tuberculosis: a population-based study in Hunan Province, China, 2009–2023
Source: Front Public Health. 2026 Mar 31;14:1755269. doi: 10.3389/fpubh.2026.1755269 (PMC13076523; doi:10.3389/fpubh.2026.1755269)
Supplement: Supplementary file 1 [file Supplementary_file_1.docx]

Supplementary Material

# Supplementary Tables

**Table S1.** Wald Test results from the age–period–cohort analysis of the reported incidence of bacteriologically confirmed pulmonary tuberculosis (PTB) in Hunan Province, 2009 to 2023

| Parameter | All | | |  | Male | | |  | Female | | |
| --- | --- | --- | --- | --- | --- | --- | --- | --- | --- | --- | --- |
|  | χ² | df | *P* |  | χ² | df | *P* |  | χ² | df | *P* |
| Net drift = 0 | 9.49 | 1 | 0.002 |  | 11.89 | 1 | 0.001 |  | 0.82 | 1 | 0.366 |
| All age deviations = 0 | 152.46 | 15 | <0.001 |  | 131.07 | 15 | <0.001 |  | 214.06 | 15 | <0.001 |
| All period deviations = 0 | 74.76 | 1 | <0.001 |  | 78.97 | 1 | <0.001 |  | 60.53 | 1 | <0.001 |
| All cohort deviations = 0 | 88.54 | 17 | <0.001 |  | 79.18 | 17 | <0.001 |  | 117.45 | 17 | <0.001 |
| All period RR = 1 | 83.93 | 2 | <0.001 |  | 90.86 | 2 | <0.001 |  | 61.03 | 2 | <0.001 |
| All cohort RR = 1 | 103.74 | 18 | <0.001 |  | 104.72 | 18 | <0.001 |  | 117.45 | 18 | <0.001 |
| All local drifts = net drift | 88.38 | 16 | <0.001 |  | 79.13 | 16 | <0.001 |  | 117.06 | 16 | <0.001 |

RR, rate ratio

**Table S2.** Local drift values of the reported incidence of bacteriologically confirmed pulmonary tuberculosis (PTB) in Hunan Province, 2009–2023, overall and stratified by sex

| Age (years) | All | |  | Male | |  | Female | |
| --- | --- | --- | --- | --- | --- | --- | --- | --- |
|  | Percent per year | 95% CI |  | Percent per year | 95% CI |  | Percent per year | 95% CI |
| 0–4 | −0.15 | (−22.10, 28.00) |  | −1.38 | (−24.93, 29.56) |  | 1.72 | (−17.94, 26.10) |
| 5–9 | 4.02 | (−15.46, 28.00) |  | 5.12 | (−16.96, 33.06) |  | 2.46 | (−13.91, 21.93) |
| 10–14 | 3.95 | (−5.36, 14.17) |  | 3.77 | (−7.93, 16.95) |  | 4.54 | (−2.36, 11.92) |
| 15–19 | 0.63 | (−2.61, 3.97) |  | −0.82 | (−4.32, 2.80) |  | 2.86 | (0.00, 5.81) |
| 20–24 | −2.85 | (−5.18, −0.45) |  | −3.73 | (−6.20, −1.20) |  | −1.39 | (−3.54, 0.81) |
| 25-29 | −6.23 | (−8.42, −3.99) |  | −6.79 | (−9.11, −4.41) |  | −5.09 | (−7.09, −3.05) |
| 30–34 | −3.39 | (−5.60, −1.13) |  | −3.74 | (−6.08, −1.35) |  | −2.48 | (−4.52, −0.40) |
| 35–39 | −3.93 | (−6.12, −1.68) |  | −4.52 | (−6.78, −2.20) |  | −2.38 | (−4.51, −0.20) |
| 40–44 | −3.89 | (−5.89, −1.85) |  | −4.58 | (−6.59, −2.53) |  | −2.12 | (−4.20, 0.01) |
| 45–49 | −4.36 | (−6.06, −2.63) |  | −4.59 | (−6.25, −2.89) |  | −3.51 | (−5.41, −1.57) |
| 50–54 | −1.62 | (−3.24, 0.03) |  | −1.77 | (−3.33, −0.18) |  | −0.46 | (−2.36, 1.48) |
| 55–59 | 0.04 | (−1.50, 1.60) |  | −0.11 | (−1.60, 1.40) |  | 0.95 | (−0.87, 2.80) |
| 60–64 | −2.33 | (−3.84, −0.79) |  | −2.20 | (−3.66, −0.71) |  | −2.17 | (−3.93, −0.38) |
| 65–59 | 0.48 | (−1.12, 2.11) |  | 0.38 | (−1.19, 1.97) |  | 1.68 | (−0.14, 3.54) |
| 70–74 | 1.97 | (0.22, 3.75) |  | 1.13 | (−0.57, 2.86) |  | 4.87 | (2.87, 6.91) |
| 75–79 | 3.05 | (0.83, 5.32) |  | 1.84 | (−0.33, 4.06) |  | 6.43 | (3.94, 8.97) |
| 80–84 | 5.77 | (1.81, 9.89) |  | 4.11 | (0.29, 8.08) |  | 10.03 | (5.37, 14.88) |

CI, confidence interval

**Table S3.** Specific outcomes of the cohort effect on the reported incidence of bacteriologically confirmed pulmonary tuberculosis (PTB) in Hunan Province, 2009–2023

| Birth cohort | All | |  | Male | |  | Female | |
| --- | --- | --- | --- | --- | --- | --- | --- | --- |
|  | RR | 95% CI |  | RR | 95% CI |  | RR | 95% CI |
| 1929–1933 | 0.80 | (0.50, 1.29) |  | 1.05 | (0.66, 1.68) |  | 0.33 | (0.19, 0.56) |
| 1934–1938 | 1.10 | (0.78, 1.54) |  | 1.30 | (0.93, 1.80) |  | 0.59 | (0.41, 0.86) |
| 1939–1943 | 1.40 | (1.05, 1.88) |  | 1.58 | (1.19, 2.10) |  | 0.86 | (0.62, 1.19) |
| 1944–1948 | 1.48 | (1.13, 1.94) |  | 1.56 | (1.20, 2.03) |  | 1.10 | (0.81, 1.49) |
| 1949–1953 | 1.70 | (1.33, 2.18) |  | 1.77 | (1.39, 2.24) |  | 1.38 | (1.04, 1.82) |
| 1954–1958 | 1.56 | (1.24, 1.95) |  | 1.62 | (1.30, 2.02) |  | 1.30 | (1.01, 1.68) |
| 1959–1963 | 1.35 | (1.09, 1.66) |  | 1.41 | (1.15, 1.73) |  | 1.11 | (0.87, 1.40) |
| 1964–1968 | 1.56 | (1.31, 1.87) |  | 1.60 | (1.34, 1.91) |  | 1.43 | (1.17, 1.74) |
| 1969–1973 | 1.14 | (0.98, 1.34) |  | 1.18 | (1.01, 1.38) |  | 1.06 | (0.89, 1.25) |
| 1974–1978 | 1.00 | (1.00, 1.00) |  | 1.00 | (1.00, 1.00) |  | 1.00 | (1.00, 1.00) |
| 1979–1983 | 0.77 | (0.64, 0.93) |  | 0.74 | (0.61, 0.90) |  | 0.85 | (0.71, 1.03) |
| 1984–1988 | 0.67 | (0.53, 0.84) |  | 0.63 | (0.50, 0.80) |  | 0.79 | (0.63, 0.98) |
| 1989–1993 | 0.54 | (0.42, 0.71) |  | 0.51 | (0.38, 0.67) |  | 0.66 | (0.52, 0.86) |
| 1994–1998 | 0.35 | (0.26, 0.48) |  | 0.31 | (0.22, 0.43) |  | 0.47 | (0.35, 0.62) |
| 1999–2003 | 0.41 | (0.29, 0.58) |  | 0.35 | (0.24, 0.50) |  | 0.58 | (0.42, 0.80) |
| 2004–2008 | 0.37 | (0.24, 0.58) |  | 0.29 | (0.18, 0.46) |  | 0.62 | (0.42, 0.91) |
| 2009–2013 | 0.60 | (0.22, 1.61) |  | 0.50 | (0.14, 1.73) |  | 0.90 | (0.43, 1.89) |
| 2014–2018 | 0.56 | (0.07, 4.57) |  | 0.47 | (0.04, 5.16) |  | 0.79 | (0.13, 4.64) |
| 2019–2023 | 0.59 | (0.04, 8.32) |  | 0.44 | (0.02, 8.38) |  | 1.07 | (0.11, 10.12) |

CI, confidence interval; RR, rate ratio
